# AI-Generated Content Disclosure and Prolonged Short-Video Engagement: A Heuristic-Systematic Risk-Trust Model Among Late-Adolescent and Emerging-Adult TikTok Users

**DOI:** 10.3390/bs16071179

**Published:** 2026-07-13

**Authors:** Yichen Xiao, Juan Du, Yidan Ding, Minyang Zhang, Yumei Jiang, Yilin Yang, Jie Liu

**Affiliations:** 1 School of Journalism and Information Communication, Huazhong University of Science and Technology, Wuhan 430074, China; m202475640@hust.edu.cn (Y.X.);; 2 School of Marxism, Huazhong University of Science and Technology, Wuhan 430074, China; 3 College of Literature, Nanjing University, Nanjing 210019, China; 522024080031@smail.nju.edu.cn; 4 School of Physical Education, Huazhong University of Science and Technology, Wuhan 430074, China; 5 College of Philosophy and Law, Shanghai Normal University, Shanghai 201418, China; 1000575132@smail.shnu.edu.cn

**Keywords:** problematic social media use, generative AI, AI-generated content disclosure, prolonged short-video engagement, digital friction, late adolescence, emerging adulthood, perceived risk, content trust, AI literacy

## Abstract

Prolonged short-video engagement in the generative-AI era may be shaped by interface cues that encourage or interrupt repeated continuation decisions in algorithmic feeds. This study examines whether AI-generated content disclosure functions as interface-level digital friction for prolonged short-video engagement among late-adolescent and emerging-adult TikTok users. Prolonged watching intention is treated as a cognitive-behavioral proximal tendency relevant to problematic social media use (PSMU), rather than as a clinical diagnosis or an emotional-disturbance outcome. Drawing on the heuristic-systematic model, we tested a dual-pathway risk-trust model in which disclosure directly affects prolonged watching intention, while perceived risk and content trust operate as mediators and AI literacy operates as a person-level boundary condition. An online between-subjects experiment was conducted with 720 valid participants aged 18–24. Disclosure had a positive direct effect on prolonged watching intention, suggesting that AI labels can initially work as salient curiosity and novelty cues. At the same time, disclosure increased perceived risk and reduced content trust, generating negative indirect pathways that constrained prolonged watching intention. AI literacy strengthened both appraisal pathways. The findings reposition AI disclosure from a mere transparency notice to a behavioral cue that can simultaneously attract attention and activate protective appraisal. They contribute to developmental and media-psychological research on prolonged engagement and PSMU-relevant mechanisms without overstating clinical implications.

## 1. Introduction

Problematic social media use (PSMU) does not usually emerge from one isolated choice to open an application. In short-form video environments, it can develop through repeated micro-decisions to remain in an algorithmic stream: watching one more clip, tolerating uncertain content, and delaying disengagement. Generative AI adds a new layer to this process. A viewer may encounter an apparently ordinary breaking-news weather clip in which a reporter warns that coastal wind speeds will reach 60 miles per hour; a moment later, the scene becomes absurd as the reporter is blown away by the wind. The clip is not real footage but an AI-generated short-form video styled as a news report. Such examples illustrate a new manifestation of social-media engagement in which synthetic audiovisual content is embedded in everyday feeds and interface cues can determine whether users pause, verify, skip, or keep watching.

This problem is closely aligned with Behavioral Sciences and with the Special Issue on problematic social media use in the background of generative AI. Generative AI lowers production costs and enables novel, visually compelling, and personalized videos, but it also blurs content origin, weakens authenticity judgments, and intensifies concerns about misinformation, privacy, and trust (Li & Yang, 2024; Hynek et al., 2025). Platforms have responded by introducing AI-generated content labels and provenance tools such as C2PA content credentials; TikTok has promoted automated labeling and reported that more than 1.3 billion videos had been marked as AI-generated content (TikTok, 2026). The central behavioral-science question is therefore not simply whether labels disclose content origin. It is whether such labels become interface-level friction that changes the psychological momentum of short-form video viewing.

Recent Behavioral Sciences articles show why the present study is framed around psychological mechanisms rather than platform retention alone. TikTok intensity is theoretically meaningful when it is linked to PSMU-relevant patterns of use, and problematic or passive use patterns are more relevant to youth mental-health outcomes than access alone (Agyapong-Opoku et al., 2025; Golding et al., 2025). Because this experiment does not diagnose PSMU, it focuses on a cognitive-behavioral proximal outcome: whether a user intends to continue watching after encountering an AI-labeled short video.

Guided by the heuristic-systematic model (Chaiken, 1980), this study treats AI-generated content disclosure as a generative-AI-specific interface cue that can alter a cognitive-behavioral continuation tendency. Because HSM is primarily an information-processing model, the outcome is prolonged watching intention rather than emotional disturbance, anxiety, loneliness, FOMO, or clinical PSMU symptoms. The analytic focus is therefore the cognitive pathway through which a label can either attract attention or prompt reflective appraisal before users decide whether to remain in or disengage from the feed.

The sample consists of legally adult TikTok users aged 18–24. This age range is framed as late adolescence and emerging adulthood within broader youth and young-people frameworks (World Health Organization, n.d.; Sawyer et al., 2018). The study therefore examines a proximal mechanism relevant to PSMU research, while explicitly leaving emotional disturbance, FOMO, isolation, and clinically defined symptoms to future work using validated PSMU instruments.

## 2. Literature Review and Theoretical Background

### 2.1. AI-Generated Labels and Source Disclosure

As an important driving force of the new round of industrial transformation, generative artificial intelligence is usually defined as a class of AI models that simulate the structure and features of input data and generate new content such as text, images, and videos according to user prompts (Banh & Strobel, 2023). In video generation, early approaches relied heavily on generative adversarial networks and autoregressive architectures to construct inter-frame relationships (Xie et al., 2025; Jan et al., 2025). These methods advanced video generation from static images to dynamic sequences, but they were often limited in temporal consistency, length, resolution, and scene generalization. More recently, diffusion models have become a dominant technical route, giving AI-generated video stronger potential in visual fidelity and scalability (Ma et al., 2025). In short-form video production, these technologies now extend across the production chain, from topic ideation to image generation, multilingual dubbing, lip synchronization, and image enhancement (J. Kim & Kim, 2024; Bigioi & Corcoran, 2023; Patel et al., 2023; J. Chen et al., 2023, 2025).

As generative AI enters short-form video production more deeply, the source of content becomes less visible. Viewers encounter finished images, sounds, and narratives, but they cannot easily infer how the content was produced. Under conditions of invisible origin, AI-generated content disclosure has become an important platform-governance tool, and its most common interface form is the AI-generated content label (Toff & Simon, 2025). Technically, this label is more than a simple textual notice. It is linked to provenance standards that bind and verify source information. Content credentials represented by C2PA aim to record the origin and processing history of media content through mechanisms such as watermarks and digital signatures, making such information verifiable (Collomosse & Parsons, 2024). Platform disclosure practices have therefore moved from relying only on creators’ self-declaration toward a combined mode of creator declaration and platform automation (El Ali et al., 2024).

AI-generated content labels have at least three functions. First, they provide transparency by disclosing the editing or production mode of content and reducing information asymmetry in the face of algorithmic opacity (Gamage et al., 2025; Toff & Simon, 2025). Second, they guide verification by giving users an entry point for judging the source of content and reducing the spread of misleading information (Feng et al., 2023). Third, they support collaborative governance by helping creators, platforms, and third-party verification mechanisms form a governance loop through machine-readable markings (Amadi et al., 2024). Existing research shows that AI labels can change users’ cognitive evaluation of content, but this effect is not one-directional. Wittenberg et al. (2025) suggested that AI labels may function as production-process explanations or as tools expected to reduce misleading content, and these roles are not identical. Li and Yang (2024) found that source information changes users’ judgments of media accuracy and reduces trust in manipulated content. Gamage et al. (2025) argued that labels may improve AI-content identification without producing stable and uniform user reactions. Overall, prior research has focused mainly on cognition and recognition. Whether these cognitive changes translate into continued watching behavior remains insufficiently explained.

### 2.2. From Prolonged Watching Intention to Problematic Social Media Use: A Proximal-Mechanism Perspective

PSMU is conceptually different from ordinary time spent online. It refers to maladaptive patterns such as preoccupation, difficulty disengaging, loss of control, compulsive checking, distress, and interference with everyday functioning. The Social Media Disorder Scale (Van den Eijnden et al., 2016), for example, assesses symptom-like dimensions such as preoccupation, tolerance, withdrawal, persistence, displacement, problems, deception, escape, and conflict. These dimensions include emotional and functional disturbance that a single post-video intention measure cannot capture. For this reason, the dependent variable in this study is not treated as PSMU itself, but as a cognitive-behavioral continuation tendency that may become relevant to PSMU when repeated across algorithmic feeds.

Prolonged watching intention (PWI) refers to a user’s subjective willingness to remain in the current short-form video stream and continue browsing subsequent or similar content after exposure to a specific video. At the platform level, continuance research concerns whether users keep using a service over time (Bhattacherjee, 2001; M. Yan et al., 2021). At the feed level, however, PWI captures a more immediate keep-scrolling decision that resembles the low-friction continuation behavior sustaining prolonged engagement (Q. Zhang et al., 2024). It is therefore positioned as a proximal mechanism: the individual response is modest, but repeated continuation choices can increase exposure duration, strengthen habit formation, and raise the difficulty of disengagement.

This proximal focus is important because developmental risk is rarely produced by one isolated viewing choice. Repeated continuation decisions can accumulate into exposure patterns that matter for self-regulation, time perception, and well-being. Recent Behavioral Sciences studies suggest that short-form video exposure, time distortion, and problematic short-form video use are meaningfully connected to self-regulatory and developmental concerns when viewing processes recur across feeds (An et al., 2025; Jiang et al., 2025; Luo et al., 2025). In a cross-sectional study of 246 students in Grades 7–12, Hoque et al. (2026) also reported negative associations between psychological well-being and several digital activities, while access-related anxiety appeared for some communication functions. These findings do not imply that one continuation decision causes harm; rather, they justify attention to the small interface-level choices through which repeated exposure and difficulty disengaging can develop.

AI-generated content makes this proximal mechanism especially visible because disclosure moves content origin into the viewing interface. A label may help users recognize that a clip was synthetically produced, but recognition does not automatically reduce engagement. Depending on the user and the content, the same label can make a video seem unusual, prompt a moment of curiosity, or introduce a reason to pause and verify (Van der Bend et al., 2023; Wittenberg et al., 2025). AI disclosure is therefore best understood as a double-edged cognitive cue: it can momentarily retain attention by making a clip stand out, while also creating reflective friction by making authenticity, risk, and trust more salient. This narrative framing keeps the manuscript focused on cognitive and behavioral mechanisms rather than treating PWI as an emotional or clinical PSMU outcome.

### 2.3. AI Disclosure as a Dual-Path HSM Cue: Attention, Risk, and Trust

The heuristic-systematic model argues that individuals do not process information through a single fixed route. They may rely on salient cues for low-effort heuristic processing while also engaging in more effortful systematic processing of message content (Chaiken, 1980). The two routes can operate in parallel, reinforce each other, or pull judgment in different directions (Maheswaran & Chaiken, 1991; Bohner et al., 1995). This framework is useful for AI disclosure because a label is both a visible interface cue and a signal about content origin. It can be noticed quickly, before the video is fully evaluated, and it can later become the basis for more deliberate judgments about provenance, accuracy, and credibility.HSM has also been adapted to explain risk perception and online information evaluation (Trumbo, 2002; K. Z. Zhang et al., 2014).

This cognitive emphasis also clarifies how audience involvement enters the model. The elaboration likelihood model highlights involvement, motivation, and ability as conditions shaping whether persuasion follows a central or peripheral route (Petty & Cacioppo, 1986; Petty et al., 1983). HSM uses a related sufficiency logic: systematic processing becomes more likely when users are motivated to reach adequate judgmental confidence and have the ability to evaluate judgment-relevant information (Chaiken & Ledgerwood, 2012). In short-form video viewing, situational involvement is often limited by rapid scrolling and low immediate stakes, but person-level ability still matters. AI literacy is therefore treated as a cue-decoding resource that can determine whether users read an AI label as ordinary metadata, a novelty signal, or a reason to verify source and content.

Short-form video viewing makes this dual process especially likely. Information flows update rapidly, dwell time is short, and users often rely on account identity, labels, visual style, subtitles, and perceived novelty for initial screening (Meinert & Krämer, 2022). In the first moments of exposure, an AI-generated content label can mark the clip as unusual and raise a small information gap about how the content was produced. Novelty, uncertainty, and incongruity can elicit exploratory behavior (Berlyne, 1960), and awareness of an information gap can motivate information seeking (Loewenstein, 1994). In a low-cost scrolling environment, such exploration may take the simple form of staying briefly rather than swiping away. Recent short-video studies likewise suggest that AI disclosure can make AI salient and increase engagement intention, even though later appraisals may reduce trust or perceived content quality (H. Chen et al., 2025; Hu & Huang, 2025).

The same disclosure cue can take on a different meaning once users move beyond the first moment of salience. Source transparency is intended to help users understand where content comes from, but in a media environment where generative AI is associated with deepfakes, hallucination, and misleading presentation, transparency may also make uncertainty more visible (Koning & Voorveld, 2025). Short-form videos are not only entertainment; they increasingly carry news, knowledge, and everyday information, so young users often hold authenticity expectations when evaluating them (Lei et al., 2026). Research on young people’s assessment of AI-generated video shows that they consider technological implementation, possible consequences, and relevant actors when judging authenticity (Lao et al., 2025). AI labels can also reduce perceived accuracy even when no obvious factual error is present (Altay & Gilardi, 2024).

Risk judgment can then extend to content trust. The label does not directly prove that a video is false, but it changes how users infer the production process. Audiences may assume greater machine involvement and weaker human oversight, which lowers default authenticity judgments (Altay & Gilardi, 2024). In news contexts, simply stating that content is AI-generated can reduce audience trust (Toff & Simon, 2025). Young users may respond by checking the publisher, comparing the clip with authoritative sources, or inspecting whether the narrative matches the image (Freeman et al., 2023). Continued watching becomes easier when the content remains sufficiently credible; once that basis is shaken, willingness to continue weakens (Lan & Tung, 2024). More broadly, short-form video retention is shaped not only by enjoyment but also by cognitive evaluation, perceived benefits, risk, and trust (Pang & Ruan, 2024; Jiang & Lau, 2021).

### 2.4. AI Literacy

AI literacy enters this process as a person-level cue-decoding capacity. It can be understood as the ability to understand, evaluate, communicate with, and apply AI systems appropriately (Long & Magerko, 2020). In youth-oriented social-media contexts, AI literacy is relevant not only to tool use but also to source evaluation, data privacy, misinformation awareness, and calibrated trust. Recent adolescent AI research in Behavioral Sciences shows that AI use and trust differ by developmental and family-related factors, with at-risk adolescent users showing heavier AI engagement and stronger trust in AI advice (Piombo et al., 2025). For short-form video viewing, AI literacy may therefore affect whether disclosure is interpreted as a mere novelty marker, a normal platform cue, or a signal that verification is needed.

AI literacy also has a constructive, not only defensive, dimension. In a mixed-method study of 117 university students, Vebibina et al. (2026) found that DeepSeek could support information access, personalized learning, efficiency, and feedback in entrepreneurship education, while the study also identified inaccuracy, dependence on technology, limited interaction, ethical risks, data-security concerns, educational gaps, and quality problems. This constructive-critical duality is important here: a user may competently exploit generative AI for learning or problem solving while also applying stricter provenance and credibility checks to synthetic media. Comprehensive AI literacy therefore combines productive tool use with calibrated skepticism, source verification, and risk evaluation (Wu et al., 2024).

However, AI literacy does not always produce a simple protective effect. Recent research has suggested an AI-literacy paradox: literacy may improve recognition ability, but it may also strengthen prior beliefs about AI and increase either automation bias or algorithm aversion (W. Kim & Ryoo, 2026). In frequent-exposure environments, higher AI literacy may intensify risk awareness after disclosure rather than simply increasing acceptance. It is therefore best understood as a boundary condition and processing resource: it shapes whether the same AI-generated content label is decoded as an ordinary interface marker, a novelty cue, or a warning that lowers trust.

## 3. Hypotheses and Model Development

### 3.1. Effects of AI-Generated Content Disclosure

Media-psychological theories of curiosity provide a specific rationale for an initial positive pathway. Berlyne (1960) proposed that novelty, uncertainty, complexity, and incongruity create arousal potential and can elicit exploratory behavior. Loewenstein’s (1994) information-gap account similarly argues that becoming aware of a gap between what one knows and what one wants to know motivates information seeking. An AI-generated-content label attached to an otherwise realistic clip can create both perceptual distinctiveness and a small explanatory gap: How was this apparently ordinary reporter generated, and what will the synthetic video show next? In a rapidly updating, low-cost feed, the least effortful exploratory response may be to defer swiping and continue watching. Together, these accounts predict a curiosity-novelty heuristic in which the label initially receives disproportionate attention before risk and trust are systematically evaluated.

In short-form video viewing, prolonged watching often occurs before deep comprehension. Users usually do not process all information in a video before deciding whether to stay; instead, they form initial judgments quickly through a small number of visible cues. From the HSM perspective, AI-generated content disclosure is a highly visible, low-cost interface cue. It can signal production attributes within a very short period and differentiate the current video from the ordinary information flow. For late-adolescent and emerging-adult users, this cue may therefore activate curiosity and novelty seeking during rapid scrolling, creating an attention-retention pathway through which disclosure increases willingness to remain in the feed.

**H1.**  *AI-generated content disclosure positively affects users’ prolonged watching intention for short-form videos.*

Disclosure, however, does more than attract attention. As AI-generated content labels become more common on platforms, users may interpret them not only as novelty prompts but also as signals of content uncertainty. HSM suggests that when a cue is associated with possible inconsistency or risk, users may move from surface judgment to more cautious systematic processing (Bohner et al., 1998). Once a video is labeled as AI-generated, users may infer algorithmic production, possible distortion, and additional verification costs (H. Jia et al., 2024; Koning & Voorveld, 2025). Because short-form videos increasingly function as informational as well as entertainment channels, such uncertainty is relevant to young users’ decisions.

**H2.**  *AI-generated content disclosure positively affects young users’ perceived risk.*

AI-generated content disclosure may also change users’ estimate of whether content is reliable. Content trust is not only an instantaneous judgment of whether a specific video is true or false; it also concerns whether users are willing to continue investing attention in the information flow to which the content belongs (Mangold et al., 2024). Disclosure may weaken this foundation not because the label proves that content is wrong, but because it changes users’ understanding of production mechanisms and responsibility. When a video is categorized as AI-generated, users may infer higher machine involvement and lower human oversight, thereby lowering default expectations of reliability (H. Jia et al., 2024). Studies in advertising and news have found that AI labels can reduce trust even when evaluations of accuracy and fairness do not necessarily decline in parallel (Toff & Simon, 2025; Grigsby et al., 2025).

**H3.**  *AI-generated content disclosure negatively affects young users’ content trust.*

### 3.2. Effects of Perceived Risk

Short-form video prolonged watching relies on a nearly frictionless decision state (Natarajan, 2024). Users often assume that continuing to scroll entails little cost, which allows autoplay and continuous recommendation to function. Once risk is perceived, this default state is disrupted. From the HSM perspective, risk is not merely a negative emotion; it means that users have shifted from rapid browsing to weighing potential consequences. At this point, users consider not only whether content is interesting, but also the costs of judgment errors, incorrect information absorption, and time consumption (Gani et al., 2025; Q. Zhang et al., 2024). This shift is important because prolonged short-video engagement depends heavily on continuous immersion and immediate feedback. When uncertainty rises, stopping, skipping, or exiting becomes a reasonable response.

**H4.**  *Perceived risk negatively affects users’ prolonged watching intention.*

Perceived risk can also raise the evidence threshold required for trust. Trust means that users are willing to grant content a basic level of credibility despite uncertainty; when risk increases, this default granting of trust contracts (Y. D. Wang & Emurian, 2005; Tseng, 2023). From the HSM perspective, once systematic processing is activated, users do not easily accept content at face value. They may re-evaluate whether the narrative is coherent, details are matched, and sources are verifiable. For young users, this re-evaluation may not be a long process of investigation; it may simply be a quick pause that produces a “do not trust yet” judgment. Research on social platforms and online services has found a stable negative relationship between perceived risk and trust (Lan & Tung, 2024; Jiang & Lau, 2021).

**H5.**  *Perceived risk negatively affects young users’ content trust.*

### 3.3. Effect of Content Trust

In HSM, systematic processing does not continue indefinitely. Once individuals believe that their judgment has reached a sufficient level of reliability, they stop further verification and redirect cognitive resources toward action; if such confidence cannot be formed, they remain alert and may discontinue contact (Chaiken & Ledgerwood, 2012). In short-form video viewing, content trust functions as the psychological signal that continued watching is acceptable. It means that users regard the current video as generally credible and are willing to reduce further verification intensity (Van Zoonen et al., 2024). This point is especially important for young users, whose viewing decisions occur in an environment of high-frequency switching and short dwell time. Whether a minimum level of trust can be established quickly directly affects whether attention remains in the current information flow or shifts to external verification.

Existing research has approached this issue through platform trust, source credibility, and information credibility, but the conclusions are broadly consistent: trust-related judgments significantly improve subsequent retention. Mobile-payment research has shown that trust promotes continued use intention (Shao et al., 2019). Sharing-economy research also regards trust as an important antecedent of continuance (Jiang & Lau, 2021). Research on Twitter similarly shows that trust in a social-media brand can enhance continued use intention (Pentina et al., 2013). In short-form video settings, TikTok studies have found that platform trust significantly improves continued use intention, and perceived credibility in health short-form videos is positively associated with post-viewing continued use tendency (Yu et al., 2025).

**H6.**  *Content trust positively affects users’ prolonged watching intention.*

### 3.4. Moderating Effects of AI Literacy

Whether an external cue triggers deeper judgment depends largely on whether users have the knowledge needed to decode it. AI literacy functions as a cue decoder. It determines whether users see a label merely as a common interface prompt or extend it into a set of meanings related to generation error, human oversight, and information consequences. Research has shown that awareness, evaluation, and ethics/risk dimensions of generative AI literacy can significantly promote information verification behavior (W. Yan et al., 2025). Studies of Generation Z students also show that AI literacy can improve diagnostic ability, prognostic ability, and risk management (Meesook et al., 2025). Thus, higher AI literacy may make young users more likely to transform a brief disclosure into an inference about content consequences.

On this basis, AI literacy may moderate the effect of AI-generated content disclosure on perceived risk. For young users with lower AI literacy, disclosure may remain a short interface cue and activate only temporary attention. For those with higher AI literacy, the label may point to automatic generation, AI hallucination, deepfakes, and verification costs, which enter judgment more quickly and amplify perceived risk.

**H7.**  *AI literacy moderates the effect of AI-generated content disclosure on young users’ perceived risk.*

AI literacy may also moderate the effect of AI-generated content disclosure on content trust. Content trust depends not only on the label itself but also on how users understand the production mechanism and responsibility allocation behind it. Young users with higher AI literacy may interpret disclosure not only as “AI was used” but also as a signal about how content was produced and checked. Consequently, after disclosure appears, they may use a higher standard to reassess whether the content deserves trust. Prior research suggests that AI literacy can change credibility evaluations of AI-generated news, but the effect is not linear because high AI literacy may reinforce either automation bias or algorithm aversion (W. Kim & Ryoo, 2026).

**H8.**  *AI literacy moderates the effect of AI-generated content disclosure on young users’ content trust.*

**Figure 1:**
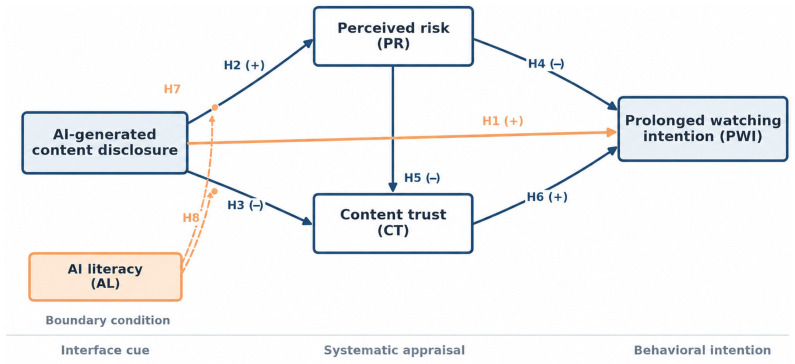
Research hypothesis model for AI disclosure, protective appraisal, and prolonged watching intention. Note. AI = AI-generated content disclosure; PR = perceived risk; CT = content trust; PWI = prolonged watching intention; AL = AI literacy. Blue solid arrows indicate the hypothesized main effects through perceived risk and content trust (H2–H6); the orange solid arrow indicates the direct effect of AI disclosure on PWI (H1); orange dashed arrows indicate the moderating effects of AI literacy (H7–H8).

Based on these hypotheses, the theoretical model is shown in Figure 1.

## 4. Materials and Methods

### 4.1. Pretest Procedure

To ensure that the stimulus materials used in the online experiment matched natural short-form video viewing, a pretest was conducted before the formal experiment. The pretest was covered by the same ethics-approved protocol and was conducted only after institutional approval was granted on 8 April 2026. The pretest did not involve label manipulation. It mainly examined two aspects: first, the stability of the materials on non-target attributes, including interest, coherence, and fluency; second, whether participants could judge that a video was AI-generated. The stimulus materials were produced with CapCut (version 4.0.0; ByteDance Ltd., Beijing, China), a video-editing tool closely connected with TikTok and suitable for generating platform-style short-form videos. Three AI-generated short-form videos on the theme of weather forecasting were produced. The three videos were kept consistent in screen structure, duration, and subtitle style and were used as candidate materials for the formal experiment.

The pretest participants were mainly international students at a university, and 81 valid responses were obtained. Participants entered the questionnaire and watched the three AI-generated weather-report videos in sequence. Before testing, they were told only that they would watch a series of short-form videos about weather forecasts. After each video, participants rated interest, coherence, and fluency on a 0–100 continuous scale and then answered whether they thought the video was generated by AI. Repeated-measures ANOVA showed that the three videos did not differ significantly in interest (F = 1.46, *p* = 0.235), coherence (F = 1.12, *p* = 0.329), or fluency (F = 0.88, *p* = 0.417), indicating that all three were suitable for a natural short-form video context. Cochran’s Q test showed a significant difference in the proportion judged to be AI-generated (Q = 11.58, *p* = 0.003). The second video had the lowest AI-recognition proportion (17.3%), lower than the first (39.5%) and third (30.9%) videos. Because the formal experiment aimed to test the effect of disclosure, a stimulus that was less likely to be identified as AI-generated in the no-label condition was preferable. Therefore, the second weather-report video was retained and two versions were made: one with an AI-generated content label and one without it.

The 17.3% recognition rate also means that 82.7% of pretest participants did not identify Video 2 as AI-generated. Non-recognition should not be equated with a direct report that the presenter was believed to be a real person, because the pretest asked only whether the clip was AI-generated, and source belief was not remeasured in the formal no-label group. Nevertheless, the result characterizes Video 2 as a high-realism, low-detection stimulus. The no-disclosure condition should therefore be interpreted as an unlabeled synthetic clip that could plausibly be processed as human-produced footage, rather than as a neutral origin-unknown baseline.

### 4.2. Online Experimental Design

The formal experiment used a single-factor, two-level, between-subjects online design. AI-generated content disclosure was the independent variable, with two conditions: a condition with an AI-generated content label and a condition without the label. The stimulus was based on the weather-report short-form video selected through the pretest. Except for whether the AI label was displayed, all other content was identical. The questionnaire was organized and translated into English through Credamo (web-based platform; version not applicable; Beijing Jianshu Technology Co., Ltd., Beijing, China; accessed April–May 2026), an online research platform that has been used in recent survey and experimental studies (Gai & Puntoni, 2021; Tang et al., 2023; H. Chen et al., 2025).

Formal recruitment was voluntary and was conducted through TikTok-based online spaces rather than through a single classroom, university, city, or province. The researchers searched short-video-related topic pages and posted a recruitment message and questionnaire link in relevant discussion pages, comment areas of popular creators, and short-video fan or interaction groups. The screening page required participants to confirm that they were aged 18–24 and had short-form video viewing experience or interest before they could continue. Because the study avoided collecting identifiable platform records and did not require province or city information, the geographic distribution of the final sample within or outside China cannot be reported; this is now acknowledged as a limitation.

The study is an original online experiment with a purpose-built stimulus and random condition assignment rather than a secondary analysis of an existing platform database. This distinction is important for the journal’s behavioral-science scope because the analysis examines how a controlled disclosure cue changes psychological appraisal and behavioral intention under experimentally manipulated viewing conditions. It is also important for the Special Issue because the outcome is interpreted as a proximal engagement mechanism relevant to problematic social media use rather than as a clinical diagnosis.

Credamo was used as the online survey and experiment platform to implement eligibility screening, random assignments, attention checks, questionnaire presentation, and small system-based compensation after valid submission. Participants were volunteers who entered through the recruitment link; they were not recruited through mandatory course participation. Compensation was modest and was provided only after completion of the consent, stimulus-viewing, and questionnaire procedures.

The questionnaire items were adapted from established scales, and the number of items, sample wording, and current reliability are reported here for transparency. Perceived risk was measured with three items adapted from Yu et al. (2025), with sample items such as “Watching this short video makes me feel a certain degree of uncertainty” and “I worry that watching this short video may have negative consequences” (Cronbach’s alpha = 0.806 in the current data). Content trust was measured with three items adapted from Appelman and Sundar (2016) and Kaňková and Matthes (2025), including accuracy, reality, and credibility judgments such as “I think the content of this short video is accurate,” “real,” and “credible” (alpha = 0.810). Prolonged watching intention was measured with three feed-level continued-watching items adapted from Bhattacherjee (2001), Hou et al. (2020), and X. Jia et al. (2023), with the final items reported below because this construct was contextually adapted most extensively (alpha = 0.812). AI literacy was measured with twelve items adapted from B. Wang et al. (2023) and W. Yan et al. (2025), including items such as “I can distinguish smart devices from non-smart devices,” “I can identify AI technologies used in the applications or products I use,” and “I can evaluate the functions and limitations of AI applications or products after using them for a period of time”; reverse-coded items captured difficulty understanding, learning, or attending to privacy and security issues in AI use (alpha = 0.940). All items were measured on a seven-point Likert scale, with 1 indicating strongly disagree and 7 indicating strongly agree.

To make the PWI adaptation transparent, the source continuance measures were treated as service- or stream-level templates rather than copied as platform-persistence items. In X. Jia et al.’s (2023) live-stream adaptation, the three source meanings were continued stream watching, choosing stream watching over television, and a reverse-coded wish to discontinue. Five contextual changes were made before data collection: (1) the referent was shifted from an information system or livestream to the short-video feed and its subsequent or similar clips; (2) the behavioral verb was shifted from continuing to use a service to continuing to watch; (3) the time frame was shifted from general future service use to an immediate post-stimulus continuation choice plus willingness to repeat that choice when similar clips recur; (4) stopping or switching to another viewing activity was used as the behavioral alternative; and (5) the reverse-coded discontinuance item was rendered as a positively worded willingness item tied to similar short videos. The final PWI items, rendered in English here, were: PWI1, ‘I intend to continue watching subsequent short videos rather than stop watching’; PWI2, ‘Compared with switching to another viewing activity (e.g., watching television), I am more inclined to continue watching short videos’; and PWI3, ‘If I encounter similar short videos in the future, I would still be willing to continue watching.’ These items were answered immediately after the assigned clip. Thus, PWI indexes a conscious feed-continuation tendency after one exposure; it does not directly record a swipe, autoplay transition, or platform-level persistence.

The statistical workflow was documented for reproducibility. The public reproduction script for data cleaning, composite-score reconstruction, condition-balance checks, correlations, regression-based moderation, and simple-slope analyses was verified under Python 3.13.5 (Python Software Foundation, Wilmington, DE, USA), using pandas 2.2.3, NumPy 2.3.5, SciPy 1.17.0, and statsmodels 0.14.6. Structural-equation and bootstrap-mediation outputs are provided as Supplementary CSV files together with the de-identified item-level dataset, variable dictionary, and public reproduction script. The public script reproduces the composite-score checks, condition counts, correlations, moderation coefficients, and simple slopes from the shared data.

The age range of youth and adolescence is not fully unified internationally. The World Health Organization commonly defines adolescence as ages 10–19, youth as ages 15–24, and young people as ages 10–24; developmental scholars have also argued that contemporary adolescence can reasonably extend to age 24 because biological maturation, identity formation, and social-role transitions often continue into the early twenties (World Health Organization, n.d.; Sawyer et al., 2018). To avoid ethical and conceptual confusion with minors, this study recruited legally adult users aged 18–24 and describes the sample as late-adolescent and emerging-adult TikTok users. This sampling decision limits direct generalization to younger adolescents, but it remains relevant to youth-oriented social-media behavior and to early mechanisms of prolonged engagement.

The study was conducted in accordance with the Declaration of Helsinki and approved by the Academic Ethics Committee of the School of Journalism and Information Communication, Huazhong University of Science and Technology (approval number HUST-SJIC-20260408; approval date: 8 April 2026). All participant-facing materials used in the survey, including the online informed-consent form and end-of-study debriefing statement, referred to this approval number and approval date. Although the ethics application materials list the General Program of the National Social Science Fund of China (grant number 22BXW056), this 2022-funded project was used only as the required administrative host project for the institutional ethics submission. It was not the start date of the present experiment, and no participant data analyzed in this manuscript were collected before ethics approval.

**Figure 2:**
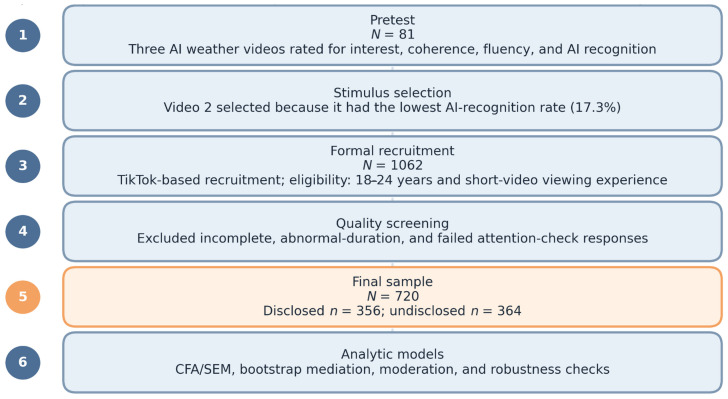
Experimental workflow and sample derivation. Note. Blue boxes indicate the procedural stages, whereas the orange box highlights the final analytic sample.

**Figure 3:**
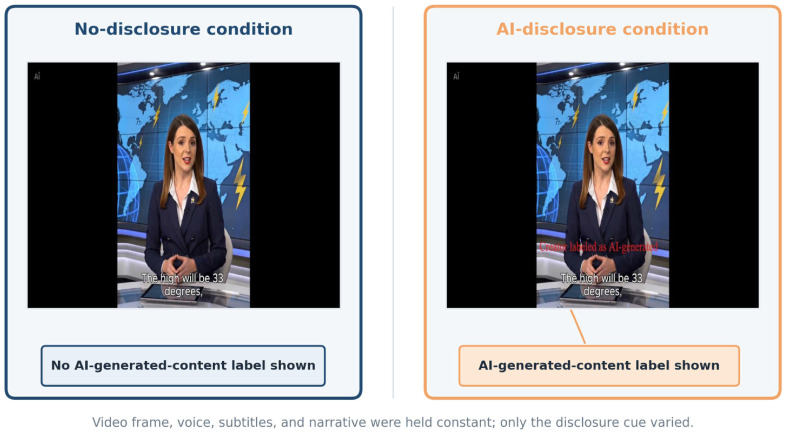
Schematic comparison of AI-generated content disclosure and no-disclosure conditions.

After screening, participants were randomly assigned to either the AI-generated content label condition or the no-label condition. They then watched the assigned video and completed the questionnaire after the video ended. A small system reward was provided after valid submission. A total of 1062 questionnaires were collected. After excluding responses with abnormal completion time, mid-process withdrawal, and failure of attention checks, 720 valid questionnaires remained: 356 in the AI-disclosure group and 364 in the no-disclosure group. The effective response rate was 67.8%. The experimental workflow is shown in Figure 2, and the disclosure manipulation is illustrated in Figure 3.

## 5. Results

### 5.1. Descriptive Statistics and Condition Checks

Among the valid participants, 53.19% were male and 46.81% were female. The proportion of males was slightly higher than that of females, which is broadly consistent with the male-leaning global user structure of TikTok reported by DataReportal (2025). In terms of educational background, 59.44% had undergraduate education, 33.19% had junior-college or technical-college education, and 4.31% had graduate education or above. Taken together, 96.94% reported some post-secondary education, indicating a highly educated and comparatively homogeneous sample. In terms of occupation, employed participants accounted for the largest group (31.25%), followed by unemployed or job-seeking participants, self-employed participants, and students. In terms of daily short-form video use, 40.69% reported 1–2 h and 29.58% reported 2 h or more, indicating that most participants were medium- to high-frequency short-video viewers. The sample characteristics are shown in Table 1.

### 5.2. Reliability and Validity

Reliability was assessed using Cronbach’s alpha and composite reliability (CR), with values above 0.70 considered acceptable (Hair et al., 2021). The results showed that Cronbach’s alpha ranged from 0.806 to 0.940 and CR ranged from 0.802 to 0.940, all exceeding the recommended threshold. This indicates satisfactory internal consistency. The reliability and validity results are shown in Table 2.

Validity was evaluated through convergent and discriminant validity. Average variance extracted (AVE) was used to assess convergent validity; all AVE values exceeded 0.50, indicating acceptable convergent validity. Discriminant validity was assessed by comparing the square root of AVE with correlations among latent constructs. The square root of AVE for each construct was greater than the absolute value of its correlations with other constructs, indicating satisfactory discriminant validity. The Pearson correlations and square roots of AVE are shown in Table 3.

### 5.3. Model Fit and Hypothesis Testing

To test the relationships among AI-generated content disclosure, perceived risk, content trust, and prolonged watching intention, a structural equation model was estimated using maximum likelihood. The results indicated good overall model fit: χ^2^ = 36.852, df = 30, *p* = 0.182, χ^2^/df = 1.228, GFI = 0.990, AGFI = 0.982, NFI = 0.986, IFI = 0.997, TLI = 0.996, CFI = 0.997, RMSEA = 0.018, RMSEA 90% CI = [0.000, 0.035], and PCLOSE = 1.000. These indicators suggest that the model fits the sample data well. Model-fit statistics are reported in Table 4.

**Figure 4:**
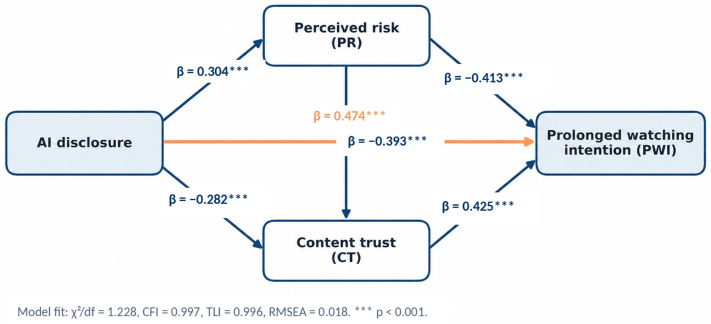
Standardized path coefficients of the structural equation model. Note. AI = AI-generated content disclosure; PR = perceived risk; CT = content trust; PWI = prolonged watching intention; *** *p* < 0.001.

The path analysis showed that AI-generated content disclosure had a significant positive effect on prolonged watching intention (β = 0.474, *p* < 0.001), supporting H1. AI-generated content disclosure significantly increased perceived risk (β = 0.304, *p* < 0.001), supporting H2. AI-generated content disclosure also significantly decreased content trust (β = −0.282, *p* < 0.001), supporting H3. Perceived risk significantly decreased prolonged watching intention (β = −0.413, *p* < 0.001), supporting H4, and significantly decreased content trust (β = −0.393, *p* < 0.001), supporting H5. Content trust significantly increased prolonged watching intention (β = 0.425, *p* < 0.001), supporting H6. The structural path coefficients are shown in Table 5 and summarized graphically in Figure 4.

### 5.4. Mediation Analysis

To test the mediation paths, bootstrap analysis with 2000 resamples was used to examine the mediating effects of perceived risk and content trust. A mediation effect was considered significant when the 95% confidence interval did not include zero. The indirect effect of AI-generated content disclosure on prolonged watching intention through perceived risk was significant (b = −0.235, 95% CI [−0.315, −0.160]). The indirect effect through content trust was also significant (b = −0.224, 95% CI [−0.317, −0.152]). The chain mediation path through perceived risk and then content trust was significant as well (b = −0.095, 95% CI [−0.131, −0.063]). The total indirect effect was negative and significant (b = −0.553, 95% CI [−0.684, −0.436]), while the direct effect of AI-generated content disclosure on prolonged watching intention remained positive and significant (b = 0.883, 95% CI [0.719, 1.049]). These findings indicate a competitive mediation pattern: disclosure directly increases initial willingness to continue watching, but it also produces reverse constraints by increasing perceived risk and reducing content trust. The results are shown in Table 6.

### 5.5. Moderation Analysis

To test H7 and H8, regression analyses were conducted to examine whether AI literacy moderated the effects of AI-generated content disclosure on perceived risk and content trust. AI-generated content disclosure was coded as 0 = no disclosure and 1 = disclosure, and AI literacy was mean-centered. In the model with perceived risk as the dependent variable, the interaction between AI-generated content disclosure and AI literacy was significant (b = 0.192, SE = 0.072, t = 2.664, *p* = 0.008), with an incremental explanatory power of ΔR^2^ = 0.008. This indicates that AI literacy strengthened the positive effect of AI-generated content disclosure on perceived risk, supporting H7. In the model with content trust as the dependent variable, the interaction was also significant (b = −0.270, SE = 0.081, t = −3.340, *p* = 0.001), with ΔR^2^ = 0.013. This indicates that AI literacy strengthened the negative effect of AI-generated content disclosure on content trust, supporting H8. After controlling for gender, education, occupation, and daily short-form video exposure, both interaction effects remained significant, suggesting that the moderation results were robust. The results are shown in Table 7.

**Figure 5:**
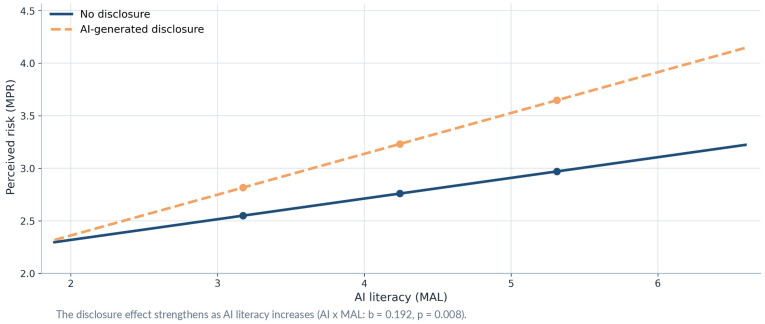
Moderating effect of AI literacy on the relationship between AI-generated content disclosure and perceived risk. Note. The vertical axis uses the perceived-risk composite score.

**Figure 6:**
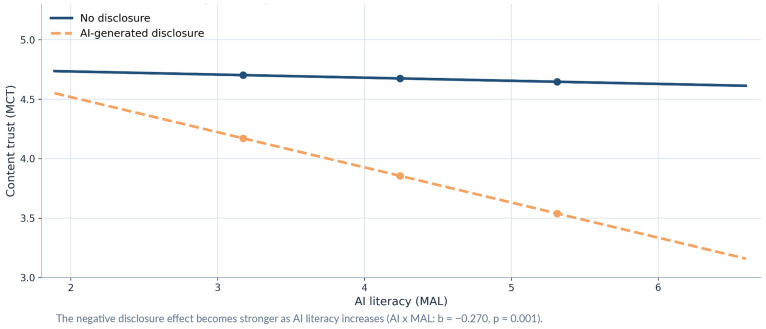
Moderating effect of AI literacy on the relationship between AI-generated content disclosure and content trust. Note. The vertical axis uses the content-trust composite score.

Simple-slope analyses further showed that, at low (−1 SD), mean, and high (+1 SD) levels of AI literacy, the effect of AI-generated content disclosure on perceived risk was positive and significant, increasing from 0.267 to 0.677. At the same three levels of AI literacy, the effect of AI-generated content disclosure on content trust was negative and significant, and the absolute effect increased from 0.532 to 1.108. These results indicate that higher AI literacy makes young users more likely to interpret AI-generated content disclosure as a risk signal and to lower their trust in the content. The simple-slope results are reported in Table 8 and visualized in Figure 5 and Figure 6.

Additional sensitivity models further addressed the observed group difference in AI literacy. When gender, education, occupation, and daily short-form video exposure were included as covariates, the AI disclosure x AI literacy interaction remained significant for perceived risk (b = 0.190, *p* = 0.009) and content trust (b = −0.284, *p* < 0.001). The direction and significance of the disclosure effects on prolonged watching intention, perceived risk, and content trust were also unchanged, suggesting that the main findings are not artifacts of measured demographic differences or baseline exposure intensity.

Because the incremental explanatory powers for the interaction terms were small, a sensitivity power analysis was added. Following the logic of F tests for multiple-regression R^2^ increase (Cohen, 1988; Faul et al., 2009), N = 720, α = 0.05, one tested predictor, and three predictors in the full interaction model provide approximately 80% power to detect a local effect of f^2^ ≈ 0.011. Using the conservative conversion f^2^ = ΔR^2^/(1 − ΔR^2^), the observed interaction effects correspond to f^2^ ≈ 0.008 for H7 and f^2^ ≈ 0.013 for H8. Thus, the H8 interaction was above the 80%-power sensitivity threshold, while the H7 interaction was statistically significant but small and should be interpreted with appropriate caution.

## 6. Discussion

### 6.1. AI Disclosure as an Attention-Retention Cue

The results show that AI-generated content disclosure positively affected prolonged watching intention among late-adolescent and emerging-adult TikTok users. This finding should not be read as evidence that young users generally prefer AI-generated content. A more cautious interpretation is that the disclosure label changed the first moment of appraisal. Consistent with Berlyne’s (1960) novelty-arousal account and Loewenstein’s (1994) information-gap account, the label made the clip stand out and invited a low-cost exploratory response: viewers could remain briefly to inspect how the synthetic content was made or what it would show next. The positive direct effect is therefore interpreted as a curiosity-novelty attention-retention heuristic rather than as stable endorsement of AI-generated media.

The low baseline recognition of the selected stimulus further qualifies this interpretation. Only 17.3% of pretest participants identified Video 2 as AI-generated, meaning that 82.7% did not identify it as synthetic. This rate does not prove that formal no-label participants explicitly believed the reporter was a real person, because that attribution was not directly measured in the formal experiment. It does, however, indicate that the no-disclosure condition involved a highly realistic synthetic clip that could plausibly be processed as human-produced footage. The direct pathway may therefore reflect both generic label novelty and the surprise produced when disclosure corrects a plausible authenticity attribution. Likewise, the risk and trust pathways may be stronger because the label violates the video’s realistic default impression.

This direct effect matters for PSMU research because problematic engagement often develops through repeated small decisions to remain in a stream. A single continuation decision is not harmful by itself, but the architecture of short-video platforms converts many such decisions into prolonged exposure. In this sense, AI disclosure can be double-edged. If the label only signals novelty, it may increase attention and support continued scrolling. If the label also provides actionable context, it may help users pause, evaluate, and disengage when content authenticity is uncertain. This interpretation directly moves the article away from a platform-retention framework and toward a behavioral-science framework concerned with proximal mechanisms of prolonged engagement.

### 6.2. Risk and Trust as Protective Digital Friction

The mediation results reveal the second side of this process. AI-generated content disclosure increased perceived risk, and perceived risk reduced prolonged watching intention. This pathway is consistent with research showing that AI labels can increase skepticism toward otherwise plausible content and that provenance cues can redirect users from effortless acceptance toward verification (Altay & Gilardi, 2024; Feng et al., 2023; Li & Yang, 2024; Wittenberg et al., 2025). In the present model, disclosure labels can operate as protective digital friction: they may interrupt the default assumption that a video corresponds to real events and remind users that the content may involve synthetic production, uncertain human oversight, or misleading presentation. The same label that first attracts attention can therefore also activate a more reflective form of appraisal.

Content trust provides the other component of this protective friction. Disclosure lowered content trust, and lower trust reduced prolonged watching intention. This finding is especially relevant for informational short videos, where credibility is part of the reason users keep watching. It extends prior work showing that AI disclosure can reduce trust in news, advertisements, or synthetic media evaluations even when the content is not necessarily false (Gamage et al., 2025; Koning & Voorveld, 2025; Toff & Simon, 2025). An AI label does not prove that content is false; however, it changes the evidentiary burden. Users may ask who produced the clip, whether the underlying information is verified, and whether the synthetic presentation changes the meaning of the message. When these questions remain unanswered, trust weakens and prolonged watching becomes less likely.

### 6.3. AI Literacy as a Developmental and Media-Psychological Boundary Condition

The moderation analysis shows that AI literacy strengthened the positive effect of disclosure on perceived risk. Users with higher AI literacy were more likely to translate a brief label into concerns about generation error, deepfakes, hallucinated details, and verification costs. This finding is consistent with AI-literacy frameworks that treat literacy as a capacity to understand, evaluate, and critically use AI systems (Long & Magerko, 2020; B. Wang et al., 2023; W. Yan et al., 2025). It does not mean that AI literacy simply makes users distrust AI. Rather, literacy can make users more sensitive to the conditions under which AI-generated content should be checked before it is accepted or further consumed.

AI literacy also strengthened the negative effect of disclosure on content trust. Higher-literacy users were less likely to equate realistic appearance with reliability. They were more likely to distinguish visual plausibility from source accountability, a distinction that is essential in generative-AI media environments. This interpretation is consistent with the AI-literacy paradox: stronger literacy can sharpen recognition and verification while also amplifying algorithm aversion or bias depending on context (W. Kim & Ryoo, 2026). It is also consistent with the constructive-critical account of AI literacy suggested by Vebibina et al. (2026): competent users may employ generative AI strategically in learning environments while applying stricter credibility checks to synthetic media in public feeds. For intervention design, AI literacy education should therefore move beyond teaching how to use AI tools. It should also train provenance awareness, calibrated trust, fact-checking habits, and strategies for disengaging from uncertain content in high-velocity feeds.

### 6.4. Strengths, Boundaries, and Alternative Explanations of the Cognitive Approach

A strength of the present HSM-based approach is that it explains how a visible platform cue can produce competing cognitive consequences: attention retention at the heuristic stage and protective appraisal at the systematic stage. This focus is appropriate for a controlled disclosure experiment because the manipulated object is an interface label and the measured outcome is a post-exposure intention. However, it is also a boundary. PSMU is not only a cognitive appraisal problem. Clinical and developmental accounts emphasize emotional disturbance, loss of control, social isolation, escape motives, FOMO, and functional impairment (Przybylski et al., 2013; Van den Eijnden et al., 2016). These experiences cannot be inferred from a single self-reported PWI scale (M. Zhang et al., 2026).

Several alternative explanations should therefore be considered. The positive direct effect may partly reflect novelty, curiosity, or surprise rather than persuasion in a strong attitudinal sense. The negative risk-trust pathways may be heightened because the weather-report genre carries unusually strong truth and civic-trust expectations. The AI-literacy moderation may partly reflect broader educational or technical familiarity despite covariate adjustment. The present design reduces but cannot eliminate these alternatives. Future research should combine HSM variables with affective measures such as FOMO, anxiety, loneliness, compulsive checking, withdrawal, and distress so that cognitive appraisal can be connected to the emotional burden experienced by users with problematic or addictive social-media patterns.

### 6.5. Implications for Generative-AI-Aware PSMU Prevention

The findings provide practical implications for prevention-oriented platform design. First, AI-generated content labels should not rely only on the phrase “AI-generated”. A minimal label may attract curiosity without giving users enough information to evaluate risk. Labels that include concise provenance information, links to verification details, or context-specific prompts may be better able to transform disclosure into reflective friction, which is consistent with research on provenance-enabled media, AI-label design, and social-media friction (Feng et al., 2023; Gamage et al., 2025; Natarajan, 2024). Second, platform governance should treat labels as behavioral interventions rather than as legal disclaimers alone (Wu et al., 2025). If labels are designed to support pausing, checking, and disengaging, they may help reduce low-reflection prolonged engagement.

Third, literacy interventions should be designed for the generative-AI media ecology in which young people actually browse. Teaching users to operate generative AI tools is insufficient. They also need to understand how synthetic audiovisual content is produced, what kinds of errors or manipulations can occur, and how to verify content without becoming overwhelmed (Long & Magerko, 2020; Vebibina et al., 2026; W. Yan et al., 2025). This implication is consistent with the Special Issue’s focus on new manifestations, mechanisms, interventions, and policy strategies for problematic social media use in the background of generative AI (L. Chen, 2026).

### 6.6. Limitations and Future Research

This study has several limitations that should be clearly recognized. First, the stimulus was a synthetic weather report, a highly specific informational genre in which audiences ordinarily expect objective truth, numerical accuracy, public-safety relevance, and institutional or civic trust. These expectations likely make perceived risk and content trust especially salient. The present findings therefore cannot be seamlessly extrapolated to entertainment, comedy, lifestyle, aesthetic-remix, or other short-video streams in which synthetic enhancement or complete AI generation may be expected, playful, or socially accepted. Future studies should cross disclosure with content genre and communicative purpose to determine whether the competitive risk-trust mechanism persists when truth evaluation is less central.

Second, the selected clip had an unusually low unlabeled AI-recognition rate: only 17.3% identified it as AI-generated, while 82.7% did not. This does not establish that participants explicitly believed the presenter was a real human, because the pretest used a binary AI-recognition question and the formal no-label group was not separately asked about perceived authorship. Nevertheless, it means that the control condition was a high-realism, low-detection synthetic baseline rather than a neutral unlabeled baseline. Disclosure may therefore have altered both label salience and perceived provenance. Future experiments should manipulate visual realism independently of disclosure and measure perceived human versus AI authorship, authenticity confidence, and recognition after exposure.

Third, the sample was educationally concentrated: 59.44% reported undergraduate education, 33.19% junior-college or technical-college education, and 4.31% graduate education or above, yielding 96.94% with some post-secondary education. Regardless of the causal relation between education and AI literacy, this homogeneity raises a restriction-of-range concern. The moderation effects remained significant after controlling for education, but statistical adjustment cannot restore the lower-literacy and lower-education segments that were sparsely represented. The buffering or risk-amplifying role of AI literacy may therefore differ in a general adolescent population with a broader educational distribution. Moreover, the participants were legally adult users aged 18–24; direct generalization to younger adolescents or minors requires age-stratified samples, parental consent, age-appropriate stimuli, and developmental measures of impulse control, emotion regulation, parental mediation, and susceptibility to platform rewards.

Fourth, the formal sample was recruited through online TikTok-related spaces and a Credamo-hosted questionnaire rather than through probability sampling. Participants were volunteers who passed eligibility and attention checks and received a small platform reward. Because the survey did not retain province, city, or IP-based location information, the geographic distribution of the final sample cannot be described. The findings should therefore be interpreted as applying to eligible online TikTok users reached through the recruitment channels rather than as a geographically representative sample within China or any specific region.

Fifth, the formal experiment used a binary manipulation of an AI-generated-content label versus no label. Real disclosures vary in wording, iconography, color, position, timing, persistence, and whether they link to provenance evidence (Henestrosa & Kimmerle, 2025). Multi-factor experiments should test whether richer explanations preserve transparency while reducing the label’s novelty-hook function and strengthening reflective verification.

Sixth, the outcome was a conscious, self-reported intention measured in a static survey immediately after one clip, not observed prolonged viewing or a validated PSMU diagnosis. Real-world problematic short-video use is sustained by continuous, passive, and sometimes mindless transitions in an infinite scroll, including sub-second stay-or-swipe decisions, autoplay, algorithmic sequencing, and time distortion (Jiang et al., 2025; Natarajan, 2024). A post hoc rating may overrepresent reflective cognition and fail to capture these micro-decisions. Future research should embed disclosure manipulations in multi-clip simulated feeds and combine self-report with swipe latency, dwell time, the number of consecutive clips viewed, pausing, skipping, rewatching, return behavior, and time-estimation error. These traces should be paired with validated measures of PSMU, compulsive checking, loss of control, FOMO, isolation, distress, and functional impairment so that the proximal mechanism identified here can be linked to clinically and developmentally meaningful outcomes.

## 7. Conclusions

This study examined how AI-generated content disclosure influences prolonged watching intention among late-adolescent and emerging-adult TikTok users. Drawing on the HSM, it tested a dual-pathway model in which perceived risk and content trust served as mediators and AI literacy served as a person-level boundary condition. The findings show that disclosure operates through competing pathways. At the heuristic stage, the label can function as a curiosity-novelty cue that increases short-term willingness to keep watching. At the systematic-appraisal stage, the same label can increase perceived risk and reduce content trust, thereby constraining prolonged watching intention through negative indirect pathways. AI literacy amplified these appraisal effects, indicating that users with stronger AI-related competencies were more likely to interpret disclosure as a reason for caution rather than as a simple novelty cue. Consistent with this construct framing, PWI is treated as a cognitive-behavioral proximal mechanism rather than as a clinical diagnosis or emotional-disturbance scale. These conclusions are bounded by a highly realistic, news-like weather stimulus, a predominantly post-secondary-educated adult sample, a volunteer online recruitment strategy without precise geographic distribution, and a self-reported post-exposure intention measure rather than observed infinite-scroll behavior. Within those boundaries, the findings support AI labels that are transparent, interpretable, and paired with literacy-oriented guidance so that users can make informed decisions about whether to remain in or disengage from algorithmic feeds.

## Figures and Tables

**Table 1:** Sample characteristics (*N* = 720).

Demographic Characteristic	Category	*n*	%
Gender	Male	383	53.19
	Female	337	46.81
Education	High school or below	22	3.06
	Junior college or technical college	239	33.19
	Undergraduate	428	59.44
	Graduate or above	31	4.31
Occupation	Student	138	19.17
	Employed	225	31.25
	Self-employed	141	19.58
	Unemployed/job seeking	146	20.28
	Other non-labor status	70	9.72
Daily short-form video	Less than 30 min	42	5.83
	30 min to 1 h	172	23.89
	1 h to 2 h	293	40.69
	2 h or more	213	29.58

**Table 2:** Reliability and validity tests. Note. PR = perceived risk; CT = content trust; PWI = prolonged watching intention; AL = AI literacy; CR = composite reliability; AVE = average variance extracted.

Dimension	Item	Standardized Loading	Cronbach’s α	CR	AVE
PR	PR1	0.778	0.806	0.810	0.588
	PR2	0.824			
	PR3	0.692			
CT	CT1	0.748	0.810	0.802	0.576
	CT2	0.801			
	CT3	0.725			
PWI	PWI1	0.762	0.812	0.814	0.593
	PWI2	0.772			
	PWI3	0.776			
AL	AL1	0.660	0.940	0.940	0.568
	AL2	0.681			
	AL3	0.771			
	AL4	0.774			
	AL5	0.788			
	AL6	0.734			
	AL7	0.758			
	AL8	0.798			
	AL9	0.779			
	AL10	0.757			
	AL11	0.765			
	AL12	0.765			

**Table 3:** Pearson correlations and square roots of AVE. Note. PR = perceived risk; CT = content trust; PWI = prolonged watching intention; AL = AI literacy. Diagonal values are square roots of AVE.

	PR	CT	PWI	AL
PR	0.767			
CT	−0.391	0.759		
PWI	−0.383	0.348	0.770	
AL	0.341	−0.223	0.107	0.754

**Table 4:** Model-fit indices.

Model	χ^2^/df	GFI	AGFI	NFI	IFI	TLI	CFI	RMSEA
SEM	1.228	0.990	0.982	0.986	0.997	0.996	0.997	0.018

**Table 5:** SEM path coefficients and hypothesis tests. Note. AI = AI-generated content disclosure; PR = perceived risk; CT = content trust; PWI = prolonged watching intention; CR = critical ratio.

Hypothesis	Path	b	SE	CR	β	*p*	Result
H1	AI → PWI	0.883	0.077	11.464	0.474	<0.001	Supported
H2	AI → PR	0.572	0.077	7.479	0.304	<0.001	Supported
H3	AI → CT	−0.599	0.085	−7.055	−0.282	<0.001	Supported
H4	PR → PWI	−0.409	0.049	−8.366	−0.413	<0.001	Supported
H5	PR → CT	−0.443	0.053	−8.434	−0.393	<0.001	Supported
H6	CT → PWI	0.373	0.046	8.127	0.425	<0.001	Supported

**Table 6:** Mediation analysis results. Note. PR = perceived risk; CT = content trust; PWI = prolonged watching intention.

Effect Path	Effect	Boot Mean	95% CI	Result
AI disclosure → PR → PWI	−0.235	−0.235	[−0.315, −0.160]	Significant
AI disclosure → CT → PWI	−0.224	−0.227	[−0.317, −0.152]	Significant
AI disclosure → PR → CT → PWI	−0.095	−0.094	[−0.131, −0.063]	Significant
Total indirect effect	−0.553	−0.555	[−0.684, −0.436]	Significant
Direct effect	0.883	0.887	[0.719, 1.049]	Significant
Total effect	0.330	0.332	[0.183, 0.483]	Significant

**Table 7:** Moderating effects of AI literacy. Note. AI literacy was mean-centered for moderation analyses; ΔR^2^ indicates incremental explanatory power of the interaction term.

Hypothesis	Dependent Variable	b	SE	t	*p*	ΔR^2^	Robustness
H7	Perceived risk	0.192	0.072	2.664	0.008	0.008	b = 0.190, *p* = 0.009
H8	Content trust	−0.270	0.081	−3.340	0.001	0.013	b = −0.284, *p* < 0.001

**Table 8:** Simple slopes of AI-generated content disclosure at different AI-literacy levels. Note. MAL = AI-literacy composite score.

Dependent Variable	AI Literacy Level	MAL Value	Simple Slope	SE	*p*	95% CI
Perceived risk	Low (−1 SD)	3.173	0.267	0.107	0.013	[0.057, 0.477]
Perceived risk	Mean	4.243	0.472	0.076	<0.001	[0.322, 0.622]
Perceived risk	High (+1 SD)	5.313	0.677	0.110	<0.001	[0.462, 0.892]
Content trust	Low (−1 SD)	3.173	−0.532	0.120	<0.001	[−0.767, −0.296]
Content trust	Mean	4.243	−0.820	0.086	<0.001	[−0.988, −0.652]
Content trust	High (+1 SD)	5.313	−1.108	0.123	<0.001	[−1.350, −0.867]

## Data Availability

The de-identified item-level dataset, analysis-ready dataset, variable dictionary, reproducibility scripts, and CSV result tables are provided as Supplementary Files. Additional materials that may contain platform-specific recruitment information or identifiable administrative records are available from the corresponding author upon reasonable request and subject to institutional review-board requirements for participant-data protection.

## Supplementary Materials

The following supporting information can be downloaded at: https://www.mdpi.com/article/10.3390/bs16071179/s1: de-identified item-level data, an analysis-ready dataset, a variable dictionary, reproducibility scripts, CSV result tables, participant-facing materials, ethics-review documentation, an ethics-timeline clarification note, and a reproducibility README. These materials document the data-cleaning decisions, variable coding, composite-score reconstruction, model outputs, randomization and sensitivity checks, and participant consent/debriefing procedures used in this study. The ethics folder includes bilingual ethics approval documents and a clarification note explaining that the 2022 grant listed in the ethics records served only as an administrative host project and that all participant data analyzed in this manuscript were collected after approval on 8 April 2026. Separate high-resolution figure files are also provided for production and verification purposes.
