# Supplementary material for: AI-Generated Content Disclosure and Prolonged Short-Video Engagement: A Heuristic-Systematic Risk-Trust Model Among Late-Adolescent and Emerging-Adult TikTok Users
Source: Behav Sci (Basel). 2026 Jul 13;16(7):1179. doi: 10.3390/bs16071179 (PMC13405702; doi:10.3390/bs16071179)
Supplement: Supplementary file 1 [file behavsci-16-01179-s001.zip › ethics/E1_Ethics_Approval_English.pdf]

Research Ethics Review Application Form

School of Journalism and Information Communication, Huazhong University of Science and Technology

I. Overview

**Ethics Approval No.**	HUST-SJIC-20260408		
**Review Date**	April 8, 2026	**Review Type**	☑ Expedited review<br>☐ Full committee review
**Type of Research Output**	☐ Project ☑ Paper (☑ Scholarly article ☐ Data report ☐ Industry-oriented paper ☐ Other)<br>☐ Experiment ☐ Other type specified: None		
**Study Title**	The Impact of AI-Generated Content Disclosure on Young Users' Engagement with Short-Form Videos: An Online Empirical Study on TikTok		
**Official English Title**	The Impact of AI-Generated Content Disclosure on Young Users' Engagement with Short-Form Videos: An Online Empirical Study on TikTok		
**Principal Investigators / Named Authors**	Zhiwu He; Minyang Zhang	**Lead Researcher Contact Number**	(+86)18071748089
**School / Department**	School of Journalism and Information Communication, Huazhong University of Science and Technology		
**Significance and Necessity of the Study (minimum 100 words)**	This study examines how disclosure that short-form video content has been generated by AI affects young users' engagement. It has clear theoretical value as well as practical significance. On the one hand, as generative AI becomes increasingly embedded in the production and circulation of short-form videos, disclosure of AI-generated content is no longer merely a voluntary statement by creators; it is gradually becoming a key mechanism in platform governance and institutional regulation. On the other hand, as an explicit cue, AI-generated content disclosure may enhance users' awareness of content provenance and strengthen their capacity for judgment, while also altering their perceptions of authenticity, credibility, and attractiveness, thereby influencing behaviors such as liking, commenting, sharing, and continued viewing. Accordingly, examining the effects of AI-generated content disclosure on young users' engagement with short-form videos will not only deepen the theoretical understanding of the relationship among AIGC communication, platform transparency, and user behavior, but also provide empirical support for improving disclosure practices and platform governance.		
**Is this a multi-institution study?**	☐ Yes ☑ No		
**Does this study involve clinical research with human or animal participants?**	☐ Yes ☑ No		

**Does this study involve biomedical experimentation on human or animal subjects?**	☐ Yes ☑ No
**Does this study involve issues related to bioethics, genetic ethics, ecological ethics, information ethics, social ethics, or related areas?**	☐ Yes ☑ No

II. Funding

**Source of Funding**	**Funding Category**	☑ National-level ☐ Provincial/Ministerial ☐ Departmental/Bureau-level ☐ University/School-level ☐ District-level ☐ Commissioned by an enterprise/public institution ☐ Self-funded ☐ Overseas-funded project
**Source of Funding**	**Name of Funded Project**	A Study of the Models and Effects of County-Level Integrated Media Participation in Rural Governance
**Source of Funding**	**Project No.**	22BXW056
**Do any collaborating parties involve foreign capital?**	☐ Yes ☑ No	
**Total Amount of Research Funding**	RMB 0	

III. Statement of Ethical Issues

**Role of Human and Animal Subjects in the Study; Study Protocol**	This project involves only human participants and no animal experimentation. The human participants are young users who voluntarily take part in the study through social media platforms. In this research, they serve as participants in an online experiment and questionnaire survey. Participants are only required to read the informed consent statement online, watch the experimental video materials, and complete the questionnaire. The study does not involve drug intervention, clinical procedures, or the collection of biological samples. Participation is entirely voluntary, and participants may discontinue or withdraw at any stage of the study without any penalty or adverse consequences.<br>Study Procedures: This study will use an online randomized experiment. Participants aged 18 to 24 will be recruited through a survey link distributed on TikTok. After opening the link, prospective participants will complete two eligibility-screening questions and, if eligible, review the informed consent form before deciding whether to participate. Those who are ineligible or decline consent will not proceed. Eligible individuals who provide consent will enter the main study and complete the initial screening items.
**Potential Harm to Humans, Animals, or Society, and Contingency Plan**	This study consists of an online questionnaire and a randomized experiment conducted via social media platforms. Overall, it qualifies as minimal-risk research, and the level of risk is, in principle, no greater than the ordinary risks participants may encounter in their

	everyday use of short-video platforms or when completing online questionnaires.

IV. Review Opinions

**Commitment by the First Author / Lead Researcher (faculty member required)**	Approved.<br><br>Committee Seal<br><br>Signature of Approver: ____________ Date: April 8, 2026
**Review Opinion of the Faculty Member's Department**	This department confirms that the information provided in this form is true and valid, and that there is no violation of laws or regulations relating to academic ethics, nor any infringement of others' intellectual property rights. Should any dispute arise, the department undertakes to cooperate fully with any investigation and follow-up handling.<br><br>Official Department Seal<br><br>Signature of Responsible Person: ____________ Date: April 8, 2026
**Approval Opinion of the Academic Ethics Committee**	Approved.<br><br>Committee Seal<br><br>Signature of Approver: ____________ Date: April 8, 2026
