# Supplementary material for: AI-Generated Content Disclosure and Prolonged Short-Video Engagement: A Heuristic-Systematic Risk-Trust Model Among Late-Adolescent and Emerging-Adult TikTok Users
Source: Behav Sci (Basel). 2026 Jul 13;16(7):1179. doi: 10.3390/bs16071179 (PMC13405702; doi:10.3390/bs16071179)
Supplement: Supplementary file 1 [file behavsci-16-01179-s001.zip › ethics/E2_Ethics_Approval_Chinese.pdf]

华中科技大学新闻与信息传播学院伦理审查申请表

一、概况

审查批准号	HUST-SJIC-20260408		
审查日期	2026 年 4 月 8 日	**审查方式**	☑ 快速审查；☐ 会议审查
研究业务类型	☐项目；☑论文（☑学术性 ☐数据报告 ☐企业性 ☐其他）<br>☐实验；☐其他类型请注明（无）		
研究题目	AI 生成披露对青年用户短视频参与度的影响研究——基于 TikTok 平台的在线实证研究		
题目的外文翻译	A Study on the Impact of AI-Generated Content Disclosure on Young Users' Engagement with Short Videos: An Online Empirical Study Based on the TikTok Platform		
全体负责人/署名作者	何志武、张民阳	**负责人联系电话**	（+86）18071748089
所在二级单位	华中科技大学新闻与信息传播学院		
研究的意义和必要性（100 字以上）	本研究围绕 AI 生成披露对青年用户短视频参与度的作用展开，具有鲜明的理论价值与现实意义。一方面，随着生成式人工智能加速进入短视频生产与传播环节，AI 生成披露已不再只是内容创作者的自发说明，而逐渐成为平台治理和制度规范中的关键机制；另一方面，AI 生成披露作为一种外显线索，既可能增强用户对内容来源的知情与判断能力，也可能改变其对内容真实性、可信度和吸引力的感知，进而影响点赞、评论、分享和持续观看等参与行为。因此，考察 AI 生成披露对青年用户短视频参与度的影响，不仅有助于深化 AIGC 传播、平台透明度与用户行为之间关系的理论解释，也能够为平台优化披露方式、提升治理有效性提供经验支持。		
本项研究是否是多单位研究		☐是：我校为 ☐ 牵头单位；☐ 参加单位	

	☑否
本项研究是否涉及人体/动物的临床研究	□是；☑否；
本项研究是否涉及人体/动物的生物医学实验的研究	□是；☑否；
本项研究是否涉及生命伦理、基因伦理、生态伦理、信息伦理、社会伦理等活动研究？	□是；☑否；

二、经费情况

经费来源	经费类型：	☑国家级□省部级□厅局级□校级/院级□区级□企事业单位委托□自筹□境外资金项目
	研究名称：	《县级融媒体参与乡村治理的模式与效果研究》
	研究编号：	22BXW056
本项研究合作各方是否有外资方？		□是；☑否
研究资助总金额	0 万元	

三、涉及问题阐述

项目中有关人体及动物在实验中的作用、实验方案	本项目仅涉及人体受试者，不涉及任何动物实验。人体受试者为通过社交媒体平台自愿参与研究的青年用户，其在本研究中的作用是作为在线实验与问卷调查的观察对象。受试者仅需在线阅读知情同意说明、观看实验视频材料并填写问卷，不涉及药物干预、临床操作、生物样本采集。同时，参与者参加本研究完全出于自愿，可在研究任何阶段中止或退出，且不会因此受到任何不利影响。<br>**实验方案：**本研究采用在线随机实验设计。研究者将通过 TikTok 平台发布问卷链接，招募年龄范围在 18-24 岁的青年用户参与研究。参与者进入链接后，首先回答两个筛选条件，不符合者则直接退出；符合者则阅读知情同意后，需要确认是否同意参加，不同意者会直接退出。以上全部通过的参与者进入正式实验，并完成基本筛查题项。
预期可能出现的对人体、动物、社会造成的伤害及其处理预案	本研究为基于社交媒体平台开展的在线问卷与随机实验，整体属于最小风险研究，其风险水平原则上不高于参与者日常使用短视频平台和填写网络问卷时可能面临的常规风险。

四、审查意见表

第一负责人/作者（需为教职工）承诺	本人承诺审查备案内容不涉及生命伦理、基因伦理、生态伦理、信息伦理、社会伦理等学术伦理问题，并同意相关论文的发表或项目的开展，<br><br>承诺人签字： 2026 年 4 月 8 日
教职工所在部门审查意见	本部门确认该表格填写情况真实有效，且不存在任何违反学术伦理相关法律法规及侵犯他人知识产权的情形。如产生争议，保证积极配合调查处理工作，<br><br>负责部门盖章<br><br>负责人签字： 2026 年 4 月 8 日
学术伦理委员会审批意见	审查通过。<br><br>委员会盖章<br><br>负责人签字： 2026 年 4 月 8 日
