# Supplementary material for: AI-Generated Content Disclosure and Prolonged Short-Video Engagement: A Heuristic-Systematic Risk-Trust Model Among Late-Adolescent and Emerging-Adult TikTok Users
Source: Behav Sci (Basel). 2026 Jul 13;16(7):1179. doi: 10.3390/bs16071179 (PMC13405702; doi:10.3390/bs16071179)
Supplement: Supplementary file 1 [file behavsci-16-01179-s001.zip › materials/M1_online_informed_consent_form.pdf]

Online Informed Consent Form

Thank you for your interest in this study. Please read the information below carefully before deciding whether to participate. If you have any questions, you may contact the researcher before proceeding. If you do not wish to participate, simply close this page; you do not need to continue.

Key Information

- Purpose: This study examines young adults' experiences of watching short-form videos and their willingness to continue viewing them.
- What you will do: Complete brief screening questions, be randomly assigned to one study condition, watch one short video, and answer a survey.
- Time: About 10-15 minutes.
- Risks: This is a minimal-risk study. Possible discomforts include mild fatigue or discomfort with some questions.
- Compensation: You will receive RMB 5 if you complete all items and successfully submit the survey.
- Voluntary participation: Your participation is entirely voluntary, and you may stop at any time.

1. Study Information

Study Title: Short-Form Video Viewing Experience Study

Institution: School of Journalism and Information Communication, Huazhong University of Science and Technology

Principal Investigator: Minyang Zhang

Researcher Contact: m202475645@hust.edu.cn

Ethics Approval: The project associated with this study has received ethics approval. Approval No.: HUST-SJIC-20260408. Approval Date: April 8, 2026.

Ethics Contact / Complaints: xwcb@hust.edu.cn

2. Why Have You Been Invited to Participate?

You may participate in this study if you meet all of the following criteria:

- You are between 18 and 24 years old.
- You have some experience with, or interest in, watching short-form videos.
- You are willing to watch a short video online and complete a survey.

3. What Is This Study About and What Will You Be Asked to Do?

This study aims to understand how young users experience short-form videos and how willing they are to continue watching after viewing them. To preserve the validity of the study, a fuller explanation will be provided after your participation.

If you agree to take part, you will usually be asked to:

- Answer a small number of screening questions to determine whether you are eligible.
- Be randomly assigned to one of the study conditions (similar to drawing lots; you cannot choose your group).

- Watch a short video.
- Complete questions about your viewing experience, evaluations of the content, your short-form video use, and your media literacy.
- Submit the survey and read the end-of-study explanation.
- Receive a small payment after successful submission.

4. How Long Will Participation Take?

Your participation will take about 10-15 minutes. The exact time may vary slightly depending on how quickly you read and respond.

5. What Are the Possible Risks or Discomforts?

This is a minimal-risk study. During participation, you may experience mild fatigue or discomfort, or you may prefer not to answer certain questions. You may stop participating at any time by closing the page. You may also skip any question you do not wish to answer.

6. Are There Any Benefits to Taking Part?

You are unlikely to receive any direct personal benefit from participating in this study. However, your participation may help researchers better understand young users' short-form video viewing experiences and may contribute to future academic research and platform governance.

7. Compensation

As a token of appreciation for your time and effort, you will receive RMB 5 if you complete all study items and successfully submit the survey. This payment is compensation for your time and inconvenience; it is not a direct benefit of the research.

The payment will be issued directly through the survey platform and is expected to be provided within 3 business days after submission. Because compensation is tied to successful submission, participants who do not successfully submit the survey will not be eligible to receive payment.

If you are unable to complete the study because of technical problems beyond your control, such as a system malfunction or network delay, please contact the researcher at m202475645@hust.edu.cn within 5 business days. We will review the circumstances and provide appropriate compensation based on your actual participation.

8. Privacy, Confidentiality, and Use of Data

The researcher will make every reasonable effort to protect your privacy and the security of your data. Your responses will be used for academic research purposes only.

Specifically:

- The research team will not disclose any information that can directly identify you in reports or publications.
- Study findings will be reported only in aggregate form and will not identify any individual participant.

- The survey platform may automatically record certain technical information for quality control or payment processing. Such information will be used only for study administration and will not be used to present the research findings.
- Study data will be accessible only to the research team and will be stored in protected accounts.

Please note that no online data transmission can be guaranteed to be completely risk-free. However, the researcher will take reasonable steps to minimize the risk of unauthorized disclosure.

9. Is Participation Voluntary?

Yes. Your participation is entirely voluntary. You may choose not to participate, or you may stop at any time without penalty or loss of any rights or benefits to which you are otherwise entitled.

10. Questions or Concerns

If you have questions about the study, please contact the researcher at m202475645@hust.edu.cn. If you have questions about your rights as a research participant, or if you wish to make a complaint, please contact xwcb@hust.edu.cn.

11. Consent Statement

Please confirm the following before continuing:

□ I confirm that I am at least 18 years old and no older than 24.

□ I have read and understood the information above.

□ I understand that my participation is voluntary and that I may stop at any time.

□ I understand that I may skip any question I do not wish to answer.

□ I understand that some details of the study will be explained more fully after participation.

□ I agree to participate in this study.

Please indicate your decision below.By selecting **“I agree to participate”** and clicking **Next**, you confirm that you have read and understood the information above and voluntarily agree to take part in this study.

If you do not wish to participate, please select **“I do not agree to participate.”** The survey will then close, and no further questions will be shown.
