# Supplementary material for: AI-Generated Content Disclosure and Prolonged Short-Video Engagement: A Heuristic-Systematic Risk-Trust Model Among Late-Adolescent and Emerging-Adult TikTok Users
Source: Behav Sci (Basel). 2026 Jul 13;16(7):1179. doi: 10.3390/bs16071179 (PMC13405702; doi:10.3390/bs16071179)
Supplement: Supplementary file 1 [file behavsci-16-01179-s001.zip › materials/M2_tiktok_recruitment_materials_public.pdf]

TikTok Recruitment Materials - English Final Version

This document provides several recruitment messages that may be used as written or adapted with only minor edits. To avoid priming participants to the specific focus of the study, a relatively neutral study title is recommended at the recruitment stage.

I. Long-Form Recruitment Copy (for posts or group announcements)

[Study Recruitment | Short-Form Video Viewing Experience Study]

Hello! We are conducting an online academic study on young adults' experiences of watching short-form videos and are inviting eligible individuals to participate.

Eligibility:

- You are between 18 and 24 years old.
- You regularly watch short-form videos or have an interest in them.

What you will do:

- Watch one short video and complete a survey.

Estimated time:

- Approximately 10 to 15 minutes.

Compensation:

- Participants who complete the study will receive RMB 5 as compensation for their time.

How to participate:

- Click the study link below. You will first see the informed consent page and can then decide whether you would like to take part.

Important note:

- Participation is completely voluntary, and you may stop at any time.
- Your responses will be used for academic research purposes only.

Researcher contact:

- Email: m202475645@hust.edu.cn

Study link:

- [public survey link removed]

II. Short Recruitment Copy (for comment sections)

Adults ages 18-24 who watch short-form videos are invited to participate in an online study on short-form video viewing experience and perceptions. The study involves watching one short video and completing a survey, takes about 10 to 15 minutes, and provides RMB 5 upon completion. Participation is voluntary, and you will see an informed consent page before deciding whether to take part. Study link: [public survey link removed]

III. Direct-Message Reply (for private-message responses)

Hello, and thank you for your interest. We are conducting an online academic study on young adults' experiences of watching short-form videos and are inviting people ages 18-24 who regularly watch short-form videos to participate. The study takes about 10 to 15 minutes and involves watching one short video and completing a survey. Participants who complete the study will receive RMB 5 as compensation for their time. If you are interested, please use the link below to review the informed consent information and decide whether you would like to participate: [public survey link removed]
