# Supplementary material for: AI-Generated Content Disclosure and Prolonged Short-Video Engagement: A Heuristic-Systematic Risk-Trust Model Among Late-Adolescent and Emerging-Adult TikTok Users
Source: Behav Sci (Basel). 2026 Jul 13;16(7):1179. doi: 10.3390/bs16071179 (PMC13405702; doi:10.3390/bs16071179)
Supplement: Supplementary file 1 [file behavsci-16-01179-s001.zip › materials/M3_end_of_study_debriefing_form.pdf]

End-of-Study Debriefing Form

Thank you for taking part in this study. This document provides a full explanation of the study's actual purpose, why some information was not fully disclosed before you participated, and what options you now have regarding your data.

Institution: School of Journalism and Information Communication, Huazhong University of Science and Technology

Principal Investigator: Minyang Zhang

Researcher Contact: m202475645@hust.edu.cn

Ethics Contact / Complaints: xwcb@hust.edu.cn

1. Official Study Title

The Effects of AI-Generated Content Disclosure on Young Adults' Willingness to Continue Watching Short-Form Videos: An Online Study on TikTok

2. What Was This Study Really About?

This study examined how 18-24-year-old users respond when a short-form video is labeled as containing AI-generated content, compared with when the same kind of video is shown without that disclosure. In particular, we were interested in participants' willingness to continue watching, their perception of risk, and their trust in the content. We also collected information about participants' familiarity with and understanding of AI.

During the study, you may have been randomly assigned to one of two conditions: one version of the video included an AI-generated-content notice, and the other did not. Aside from the presence or absence of that notice, the two video versions were held constant in length, resolution, and synchronized audio.

3. Why Were You Not Given a Full Explanation at the Start?

If participants had been told in advance that the study specifically focused on whether AI-generated-content disclosure affects willingness to continue watching, they might have paid unusual attention to the label or adjusted their answers deliberately. That, in turn, could have distorted the results.

For that reason, we used a more neutral study title and general description at the beginning. This incomplete disclosure was used only to preserve the validity of the study and better approximate a natural viewing situation; it was not used to expose you to any hidden or additional risk.

4. Did This Study Involve Any Additional Risk?

No. This was a minimal-risk study. It did not involve medication, clinical treatment, biological sample collection, or any other high-risk procedure. Your participation was limited to watching a short video and completing a questionnaire.

5. What If You Do Not Want Us to Use Your Data?

Now that you know the study's actual purpose, you may decide that you do not want your data to be included in the research. If that is your preference, please contact the researcher by email at m202475645@hust.edu.cn within 14 business days after your participation. There will be no penalty for making this request.

If your data can still be located and have not yet been irreversibly anonymized and combined with other participants' data, we will honor your request to withdraw your data from the study.

6. Privacy and Confidentiality

Your data will be handled in the manner described in the consent materials. To the greatest extent possible, the research team will analyze the data in de-identified form, and the results will be reported only in aggregate. No information that directly identifies you will be disclosed in publications, presentations, or other research outputs.

7. Thank You and Contact Information

Thank you for supporting this research. Your participation is valuable for understanding how AI-generated-content labels shape audience responses to short-form video. If you have questions about the study, please contact the researcher below.

Researcher: Minyang Zhang

Email: m202475645@hust.edu.cn

Ethics Contact / Complaints: xwcb@hust.edu.cn
