# Supplementary material for: AI-Generated Content Disclosure and Prolonged Short-Video Engagement: A Heuristic-Systematic Risk-Trust Model Among Late-Adolescent and Emerging-Adult TikTok Users
Source: Behav Sci (Basel). 2026 Jul 13;16(7):1179. doi: 10.3390/bs16071179 (PMC13405702; doi:10.3390/bs16071179)
Supplement: Supplementary file 1 [file behavsci-16-01179-s001.zip › materials/M4_final_questionnaire_pages_public.pdf]

Short-Form Video Viewing Experience Study

Welcome, and thank you for your interest in this academic study.

We are conducting a study about how young adults experience short-form video content. Please answer the two brief questions below so we can determine whether this study is a good fit for you.

If you are eligible, you will be taken to the informed consent page, where you can read more about the study and decide whether you would like to participate. If you are not eligible, the survey will end automatically. Completing these screening questions does not obligate you to take part in the study.

If you choose to participate, the full study will take about 10–15 minutes to complete. Eligible participants who complete the study will receive RMB 5. Participation is entirely voluntary, and you may stop at any time by closing the page.

To protect the quality of the study, the description on this first page is intentionally brief. A fuller explanation will be provided after participation.

* Are you currently 18 to 24 years old?

- ○ Yes
- ○ No

条件：No 已选定 跳至：提交问卷 跳转URL：

* Have you watched short-form videos before (for example, on TikTok, Instagram Reels, or YouTube Shorts)?

- ○ Yes
- ○ No

条件：No 已选定 跳至：提交问卷 跳转URL：

*

Online Informed Consent Form

Thank you for your interest in this study. Please read the information below carefully before deciding whether to participate. If you have any questions, you may contact the researcher before proceeding. If you do not wish to participate, simply close this page; you do not need to continue.

Key Information

· Purpose: This study examines young adults' experiences of watching short-form videos and their willingness to continue viewing them.
· What you will do: Complete brief screening questions, be randomly assigned to one study condition, watch one short video, and answer a survey.
· Time: About 10-15 minutes.
· Risks: This is a minimal-risk study. Possible discomforts include mild fatigue or discomfort with some questions.
· Compensation: You will receive RMB 5 if you complete all items and successfully submit the survey.
· Voluntary participation: Your participation is entirely voluntary, and you may stop at any time.

1. Study Information

Study Title: Short-Form Video Viewing Experience Study
Institution: School of Journalism and Information Communication, Huazhong University of Science and Technology
Principal Investigator: Minyang Zhang
Researcher Contact: m202475645@hust.edu.cn
Ethics Approval: The project associated with this study has received ethics approval. Approval No.: HUST-SJIC-20260408. Approval Date: April 8, 2026.
Ethics Contact / Complaints: xwcb@hust.edu.cn

2. Why Have You Been Invited to Participate?

You may participate in this study if you meet all of the following criteria:
· You are between 18 and 24 years old.
· You have some experience with, or interest in, watching short-form videos.
· You are willing to watch a short video online and complete a survey.

· You are willing to watch a short video online and complete a survey.

3. What Is This Study About and What Will You Be Asked to Do?

This study aims to understand how young users experience short-form videos and how willing they are to continue watching after viewing them. To preserve the validity of the study, a fuller explanation will be provided after your participation.

If you agree to take part, you will usually be asked to:

· Answer a small number of screening questions to determine whether you are eligible.

· Be randomly assigned to one of the study conditions (similar to drawing lots; you cannot choose your group).

· Watch a short video.

· Complete questions about your viewing experience, evaluations of the content, your short-form video use, and your media literacy.

· Submit the survey and read the end-of-study explanation.

· Receive a small payment after successful submission.

4. How Long Will Participation Take?

Your participation will take about 10-15 minutes. The exact time may vary slightly depending on how quickly you read and respond.

5. What Are the Possible Risks or Discomforts?

This is a minimal-risk study. During participation, you may experience mild fatigue or discomfort, or you may prefer not to answer certain questions. You may stop participating at any time by closing the page. You may also skip any question you do not wish to answer.

6. Are There Any Benefits to Taking Part?

You are unlikely to receive any direct personal benefit from participating in this study. However, your participation may help researchers better understand young users' short-form video viewing experiences and may contribute to future academic research and platform governance.

7. Compensation

As a token of appreciation for your time and effort, you will receive RMB 5 if you complete all study items and successfully submit the survey. This payment is compensation for your time and inconvenience; it is not a direct benefit of the research.

The payment will be issued directly through the survey platform and is expected to be provided within 3 business days after submission. Because compensation is tied to successful submission, participants who do not successfully submit the survey will not be eligible to receive payment.

If you are unable to complete the study because of technical problems beyond your control, such as a system malfunction or network delay, please contact the researcher at m202475645@hust.edu.cn within 5 business days. We will review the circumstances and provide appropriate compensation based on your actual participation.

8. Privacy, Confidentiality, and Use of Data

The researcher will make every reasonable effort to protect your privacy and the security of your data. Your responses will be used for academic research purposes only.

Specifically:

· The research team will not disclose any information that can directly identify you in reports or publications.

· Study findings will be reported only in aggregate form and will not identify any individual participant.

· The survey platform may automatically record certain technical information for quality control or payment processing. Such information will be used only for study administration and will not be used to present the research findings.

· Study data will be accessible only to the research team and will be stored in protected accounts.

Please note that no online data transmission can be guaranteed to be completely risk-free. However, the researcher will take reasonable steps to minimize the risk of unauthorized disclosure.

9. Is Participation Voluntary?

Yes. Your participation is entirely voluntary. You may choose not to participate, or you may stop at any time without penalty or loss of any rights or benefits to which you are otherwise entitled.

10. Questions or Concerns

If you have questions about the study, please contact the researcher at m202475645@hust.edu.cn. If you have questions about your rights as a research participant, or if you wish to make a complaint, please contact xwcb@hust.edu.cn.

11. Consent Statement

Please confirm the following before continuing:

· I confirm that I am at least 18 years old and no older than 24.

· I have read and understood the information above.

· I understand that my participation is voluntary and that I may stop at any time.

· I understand that I may skip any question I do not wish to answer.

· I understand that some details of the study will be explained more fully after participation.

· I agree to participate in this study.

* Please indicate your decision below.By selecting **“I agree to participate”** and clicking **Next**, you confirm

[illegible] that you have read and understood the information above and voluntarily agree to take part in this study.

If you do not wish to participate, please select **"I do not agree to participate."** The survey will then close, and no further questions will be shown.

- I agree to participate
- I do not agree to participate

条件：I do not agree to participate 已选定 跳至：提交问卷 跳转URL：

* The short video for this study appears below on this page.Please watch the video carefully and as you normally would on a short-form video platform. After the video ends, you will be asked several questions about your reactions to the video and your viewing experience.

There are no right or wrong answers. We are interested in your own immediate impressions. Please watch the video through to the end before continuing.When you are ready, please begin watching the video below.

更换视频 删除视频

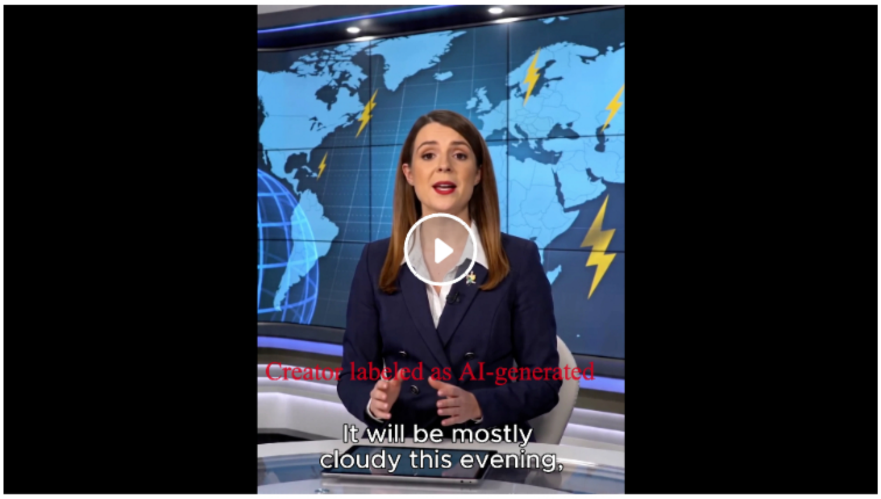

* The short video for this study appears below on this page.Please watch the video carefully and as you normally would on a short-form video platform. After the video ends, you will be asked several questions about your reactions to the video and your viewing experience.

There are no right or wrong answers. We are interested in your own immediate impressions. Please watch the video through to the end before continuing.When you are ready, please begin watching the video below.

更换视频 删除视频

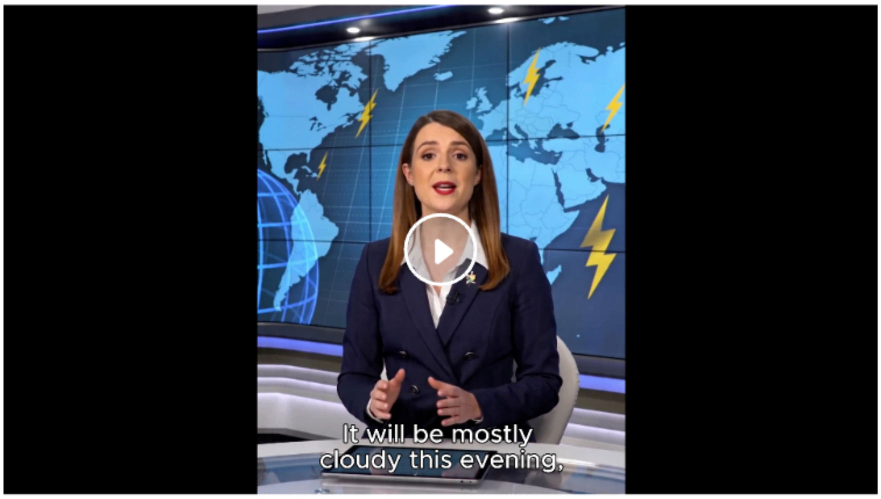

* Thank you for watching the video. The questions below ask about your reactions to the video you just watched and about your general experience with short-form videos and AI tools. There are no right or wrong answers. Please answer honestly based on your own views and first impressions. Unless otherwise indicated, you may skip any question you would prefer not to answer.

Response scale for Questions: 1 = Strongly disagree; 2 = Disagree; 3 = Somewhat disagree; 4 = Neither agree nor disagree; 5 = Somewhat agree; 6 = Agree; 7 = Strongly agree.

*

Section A. Your Reactions After Watching the Video

Q1* I would like to continue watching short-form videos after this one rather than stop now.

Strongly disagree Strongly agree

1 2 3 4 5 6 7

Q2* Instead of switching to another viewing option (such as television), I would be more likely to continue watching short-form videos.

Strongly disagree Strongly agree

1 2 3 4 5 6 7

Q3* If I came across similar short-form videos in the future, I would still be willing to keep watching them.

Strongly disagree Strongly agree

1 2 3 4 5 6 7

*

Section B. Your Assessment of the Video

Q4* The content of this video is accurate.

Strongly disagree Strongly agree

1 2 3 4 5 6 7

Q5* The content of this video feels authentic.

Strongly disagree Strongly agree

1 2 3 4 5 6 7

Q6* The content of this video is believable.

Strongly disagree Strongly agree

1 2 3 4 5 6 7

*

Section C. Did the Video Raise Any Concerns?

Q7* This video leaves me feeling somewhat uncertain.

Strongly disagree Strongly agree

1 2 3 4 5 6 7

Q8* I am concerned that this video could lead to negative consequences.

Strongly disagree Strongly agree

1 2 3 4 5 6 7

Q9* The potential risks associated with this video are difficult to predict.

Strongly disagree Strongly agree

1 2 3 4 5 6 7

*

Section D. Your Experience with AI Tools

In this section, 'AI tools' refers broadly to AI applications, platforms, or smart products that you may use in everyday life.

Q10* I can tell the difference between devices that use AI and those that do not.

Strongly disagree Strongly agree

1 2 3 4 5 6 7

Q11* I do not understand how AI tools can help me.

Strongly disagree Strongly agree

1 2 3 4 5 6 7

Q12* I can recognize which AI technologies are built into the apps and products I use.

Strongly disagree Strongly agree

1 2 3 4 5 6 7

Q13* I can use AI tools effectively to help with everyday tasks.

Strongly disagree Strongly agree

1 2 3 4 5 6 7

Q14* I usually find it difficult to learn how to use new AI tools.

Strongly disagree Strongly agree

1 2 3 4 5 6 7

Q15* I can use AI tools to work more efficiently.

Strongly disagree Strongly agree

1 2 3 4 5 6 7

Q16* After using an AI tool for a while, I can judge what it can do well and where its limits are.

Strongly disagree Strongly agree

1 2 3 4 5 6 7

Q17* I can choose the most suitable option from the different suggestions offered by an intelligent agent.

Strongly disagree Strongly agree

1 2 3 4 5 6 7

Q18* When I need to complete a specific task, I can choose the most suitable AI tool from several options.

Strongly disagree Strongly agree

1 2 3 4 5 6 7

Q19* When I use AI tools, I follow ethical principles.

Strongly disagree Strongly agree

1 2 3 4 5 6 7

Q20* When I use AI tools, I do not pay attention to privacy or information security.

Strongly disagree Strongly agree

1 2 3 4 5 6 7

Q21* I stay alert to the potential misuse of AI technologies.

Strongly disagree Strongly agree

1 2 3 4 5 6 7

Q22* To help us ensure data quality, please select 4 for this item.

Strongly disagree Strongly agree

1 2 3 4 5 6 7

*

Section E. About You

These questions are used only to describe the sample.

Q23* Which of the following best describes your gender?

- Man
- Woman
- Prefer not to say

Q24* What is the highest level of education you have completed?

[illegible]

- High school or below
- Vocational / technical college or junior college
- Bachelor's degree
- Graduate degree or above

Q25* Which of the following best describes your current employment status?

- Student
- Employed
- Self-employed
- Unemployed / looking for work
- Not currently in the labor force (e.g., medical leave, homemaking, caregiving)

Q26* On a typical day, how much time do you spend watching short-form videos?

- Less than 30 minutes
- 30 minutes to under 1 hour
- 1 hour to under 2 hours
- 2 hours or more

*

End-of-Study Debriefing Form

Thank you for taking part in this study. This document provides a full explanation of the study's actual purpose, why some information was not fully disclosed before you participated, and what options you now have regarding your data.

Institution: School of Journalism and Information Communication, Huazhong University of Science and Technology
Principal Investigator: Minyang Zhang
Researcher Contact: m202475645@hust.edu.cn
Ethics Contact / Complaints: xwcb@hust.edu.cn

1. Official Study Title

The Effects of AI-Generated Content Disclosure on Young Adults' Willingness to Continue Watching Short-Form Videos: An Online Study on TikTok

2. What Was This Study Really About?

This study examined how 18-24-year-old users respond when a short-form video is labeled as containing AI-generated content, compared with when the same kind of video is shown without that disclosure. In particular, we were interested in participants' willingness to continue watching, their perception of risk, and their trust in the content. We also collected information about participants' familiarity with and understanding of AI.

During the study, you may have been randomly assigned to one of two conditions: one version of the video included an AI-generated-content notice, and the other did not. Aside from the presence or absence of that notice, the two video versions were held constant in length, resolution, and synchronized audio.

3. Why Were You Not Given a Full Explanation at the Start?

If participants had been told in advance that the study specifically focused on whether AI-generated-content disclosure affects willingness to continue watching, they might have paid unusual attention to the label or adjusted their answers deliberately. That, in turn, could have distorted the results.

For that reason, we used a more neutral study title and general description at the beginning. This incomplete disclosure was used only to preserve the validity of the study and better approximate a natural viewing situation; it was not used to expose you to any hidden or additional risk.

4. Did This Study Involve Any Additional Risk?

No. This was a minimal-risk study. It did not involve medication, clinical treatment, biological sample collection, or any other high-risk procedure. Your participation was limited to watching a short video and completing a questionnaire.

5. What If You Do Not Want Us to Use Your Data?

Now that you know the study's actual purpose, you may decide that you do not want your data to be included in the research. If that is your preference, please contact the researcher by email at m202475645@hust.edu.cn within 14 business days after your participation. There will be no penalty for making this request.

If your data can still be located and have not yet been irreversibly anonymized and combined with other participants' data, we will honor your request to withdraw your data from the study.

6. Privacy and Confidentiality

Your data will be handled in the manner described in the consent materials. To the greatest extent possible, the research team will analyze the data in de-identified form, and the results will be reported only in aggregate. No information that directly identifies you will be disclosed in publications, presentations, or other research outputs.

7. Thank You and Contact Information

Thank you for supporting this research. Your participation is valuable for understanding how AI-generated-content labels shape audience responses to short-form video. If you have questions about the study, please contact the researcher below.

Researcher: Minyang Zhang
Email: m202475645@hust.edu.cn
Ethics Contact / Complaints: xwcb@hust.edu.cn

* Now that you have read the information above and learned the full purpose of this study, please indicate whether you would like your responses to be included in this research project.Please **select one option** below:

○ I have read and understood the debriefing information, and I agree that my responses may be used for research purposes.

○ I have read and understood the debriefing information, and I do not want my responses to be used. Please withdraw my data from this study.

条件: I have read and understood the debriefin... 跳至: 提交问卷 跳转URL:

条件: I have read and understood the debriefin... 跳至: If you later decide that you do not wan...

* If you later decide that you do not want your data to be used, please contact the researcher within **14 business days** at **m202475645@hust.edu.cn**. Your data can be withdrawn to the extent possible, provided that your responses can still be identified and have not yet been irreversibly anonymized or aggregated.
